# Clearance of human papillomavirus related anal condylomas after oral and endorectal multistrain probiotic supplementation in an HIV positive male: A case report

**DOI:** 10.1097/MD.0000000000010329

**Published:** 2018-04-20

**Authors:** Giancarlo Ceccarelli, Eugenio Nelson Cavallari, Stefano Savinelli, Luigi Bianchi, Alessandra Pierangeli, Francesco Vullo, Antonio Ciardi, Gabriella D’ettorre

**Affiliations:** a Department of Public Health and Infectious Diseases; b Department of Molecular Medicine, Laboratory of Virology; c Department of Radiological, Oncological and Pathology Sciences, “Sapienza” University of Rome, Italy.

**Keywords:** Bowen's disease, condylomas, HIV, HPV, papilloma, probiotics, squamocellular intraepithelial carcinoma

## Abstract

**Introduction:**

Here we present the case of a 56-year-old human immunodeficiency virus (HIV)-infected man with multiple anal condylomas and positivity for human papilloma virus (HPV) 18 on anal brushing. Biopsies of the anal mucosa led to the diagnosis of Bowen's disease and a subsequent pelvic magnetic resonance imaging (MRI) scan evidenced multiple reactive lymphoadenopathies and large intra-anal condylomas. The patient was treated with a complete excision of Bowen's lesion and with a 4 months course of supplementation with a high concentration multistrain probiotic formulation administered orally and by rectal instillation with the purpose to reduce local inflammation and to enhance local mucosal immunity.

**Conclusion:**

An MRI performed at the end of the supplementation period evidenced the clearance of the anal condylomas previously described and no evidence of residual lymphadenopathies. Trials are therefore required to confirm this therapeutic possibility and for a better understanding of the mechanisms by which this specific probiotic formulation interacts with local epithelium when administered by the anal route.

## 1 Introduction

The human immunodeficiency virus (HIV)-infected population shows a higher prevalence of human papilloma virus (HPV) infection and higher rates of HPV related dysplastic lesions in comparison to the general population. Recent studies suggest that the HIV related immune suppression and the interaction between HIV and HPV themselves could be responsible for the increased risk of HPV related malignancies in this population.^[1–4]^ Moreover, while HIV-infected individuals almost invariably show alterations of the normal composition of the gut microbial flora,^[5]^ evidences from literature highlight that a balanced microbiota could exert a role in the clearance of HPV infection thus preventing the risk of HPV related carcinogenesis.^[6]^ In HIV positive patients, gut dysbiosis is associated with impairment of local immune system ^[7–9]^ and in this scenario any corrective action may contribute to reduce the persistence of HPV infection and subsequently to the onset of epithelial dysplasia.

The use of high doses of oral multistrain probiotics has been investigated as a promising tool for the treatment of gut dysbiosis and the restoration of gut mucosal immunity in HIV-infected people.^[10]^ Data about the effects of local rectal enema with probiotics on anal HPV disease in HIV-infected individuals are unavailable.

## 2 Case presentation

We present the case of a 56-year-old homosexual man who was diagnosed with HIV infection in 1998 and which progressed to AIDS in the same year (CD4 nadir 6 cell/μL associated to pneumonia due to *Pneumocystis jirovecii*, cervical Herpes Zoster, and disseminated nontuberculous mycobacterial infection). During the follow-up, the patient continuously showed poor adherence to antiretrovirals and required numerous therapy switch due to virological failure. His medical records showed other pathologic conditions such as thyroid nodular disease, arterial hypertension, syphilis, recurrent anal warts repeatedly treated with diathermocoagulation and persistent positivity of HPV 18 in anal brushing.

**Figure 1:**
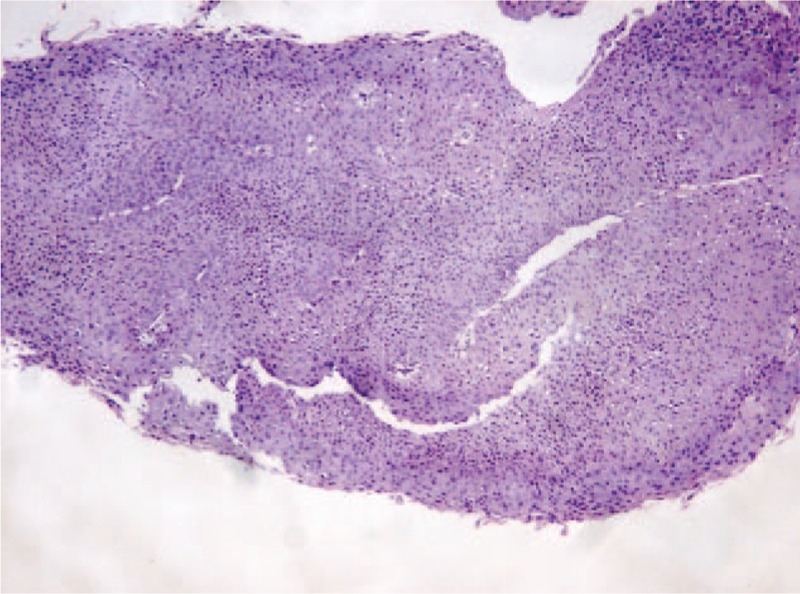
Histology section of Bowen's disease lesion.

**Figure 2:**
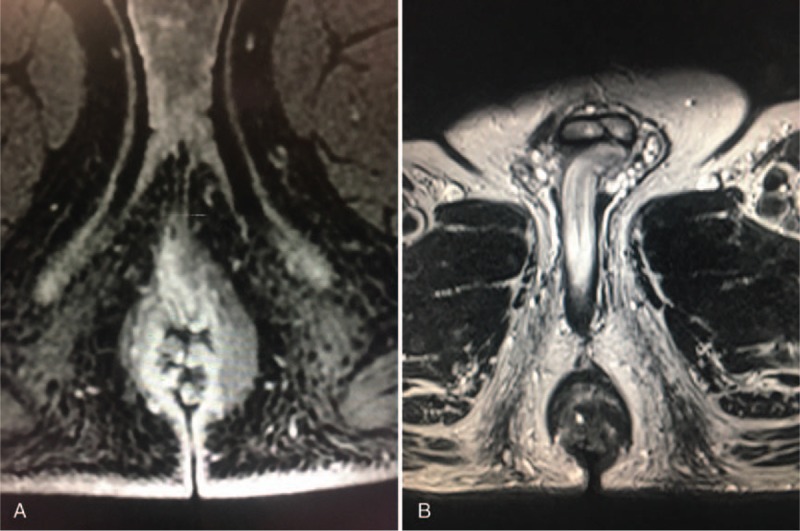
Pelvic MRI scan performed before (A) and after (B) supplementation with rectal and oral probiotics. MRI = magnetic resonance imaging.

On May 2016, during the periodic follow-up with high resolution anoscopy (HRA), it was highlighted the presence of multiple intra anal condylomas. Bioptic samples of the anal mucosa led to the diagnosis of squamocellular intraepithelial carcinoma (Bowen's disease, anal intraepithelial neoplasia AIN3) (Fig. 1), with an incomplete excision of the lesion. A pelvic MRI scan evidenced granulation tissue in the location of the recent biopsy, without radiological evidence of residual pathological tissue. The MRI also evidenced multiple condylomas of the anal canal, reactive mesorectal and pelvic lymphadenopathies, reactive lymphnodes along the sigmoidal vessels, and thickening of the mesorectum (Fig. 2A). At the time of anal Bowen's disease diagnosis, the patient was clinically asymptomatic and showed a good immune-virological status with a CD4 count of 818 cells/μL (28.27%) and HIV-RNA persistently undetectable since 2014.

Given the fact that Bowen's disease is considered a high grade dysplasia (AIN3) with a low risk of invasion (around 5%), we adopted a conservative approach undergoing a complete excision of the lesion and, once achieved, proposing to the patient a course of supplementation with a multistrain probiotic with daily endorectal instillation of 900 billion live bacteria and daily oral administration of 3 sachets, each containing 450 billion live bacteria (probiotic composition: *Lactobacillus plantarum* DSM 24730, *Streptococcus thermophilus* DSM 24731, *Bifidobacterium breve* DSM 24732, *Lactobacillus paracasei* DSM 24733, *Lactobacillus delbrueckii* subsp, *bulgaricus* DSM 24734, *Lactobacillus acidophilus* DSM 24735, *Bifidobacterium longum* DSM 24736, and *Bifidobacterium infantis* DSM 24737; commercially available as Vivomixx in Europe, Visbiome in USA and DeSimone Formulation in Korea), with the aim to reduce local inflammation and to rebalance local microbiota, thus possibly restoring in site mucosal immunity.

After 4 months of supplementation, a new pelvic MRI scan evidenced the spot of the previous biopsy but no evidence of mesorectal, iliac, and pelvic lymphadenopathies. Moreover, clearance of the anal condylomas previously described was noted (Fig. 2B) despite HPV18 DNA was still detectable.

Six months later, the patient underwent a new HRA that showed no evidence of condyloma.

## 3 Discussion

This case report suggests a potential role for this specific probiotic formulation in promoting the regression of anal HPV related lesions in HIV-infected individuals. The effects of a number of oral probiotic formulation on HPV associated alterations have been mainly studied in the female genital tract; in a prospective controlled pilot study, women with HPV associated low-grade squamous intraepithelial lesions were randomized for probiotic supplementation: the treated group showed a higher chance of clearing HPV infection (29% vs 19%, *P = *.41) and higher rates of clearance of HPV associated cytological abnormalities (60% vs 31%; *P = *.05) in comparison to the control group.^[11]^ Other evidences show that a reduced amount of *Lactobacillus spp.* combined with an increased diversity of the vaginal microbiota could be involved in HPV acquisition and persistence and in the development of dysplastic cervical lesions^[12,13]^; moreover, in vitro studies demonstrated a direct cytotoxic effect of certain vaginal microbial strains on HPV-infected cells and an inhibition of the expression of HPV E6 and E7 oncogenes, which may prevent malignant transformation in individuals harboring high-risk HPV genotypes.^[14–16]^

Based on the findings of the mentioned gynecological studies, in which the prescription of probiotic supplementation seems related with the regression of HPV related female genital tract lesions, its our opinion that probiotics could also exerted a therapeutic role in our patient. In fact, despite at the moment there are no similar direct evidences for anal condylomatosis, we believe that the effects of probiotic strains (described for female genital tract) are also possible in the anal district, as observed in this case.

Moreover in HIV positive patients, dysbiosis of the gut microbiota largely contributes to the impairment of mucosal immunity and could facilitate HPV persistence and the subsequent epithelial transformation. Given that spontaneous regression of multiple large condylomas, such as those observed in our patient, is not frequently seen among HIV-infected patients, we hypothesize a beneficial role exerted by this specific probiotic formulation in the clearance of HPV related lesions.

Finally, data on the effects of probiotic rectal enema on anal HPV infection in HIV positive males are lacking. Given the extremely distal localization of the HPV related lesions of our patient, we decided to use, together with oral administration, rectal enemas of probiotics to maximize the amount of bacterial cells that would reach the anal canal to effectively interact with the local epithelium.

## 4 Conclusions

Growing evidences show the potential beneficial effect of probiotics on HPV related disease, also in anal district. In particular, in HIV-infected individuals, due to the impairment of the immune response and the loss of gut intraepithelial lymphocytes, lower rates of HPV clearance are observed and consequently spontaneous resolution of multiple large anal condylomas is rare among this population. For this reason, the positive results of our preliminary experience support the hypothesis of a direct efficacy of oral and rectal multistrain probiotic administration in the treatment of anal condylomatosis in HIV-infected patients.

## Author contributions

**Conceptualization:** Giancarlo Ceccarelli, Gabriella d’Ettorre.

**Data curation:** Giancarlo Ceccarelli, Stefano Savinelli, Luigi Bianchi, Alessandra Pierangeli, Francesco Vullo, Antonio Ciardi, Gabriella d’Ettorre.

**Formal analysis:** Francesco Vullo.

**Investigation:** Stefano Savinelli, Luigi Bianchi, Alessandra Pierangeli, Francesco Vullo.

**Methodology:** Giancarlo Ceccarelli, Alessandra Pierangeli.

**Resources:** Stefano Savinelli, Alessandra Pierangeli.

**Supervision:** Antonio Ciardi, Gabriella d’Ettorre.

**Validation:** Alessandra Pierangeli, Gabriella d’Ettorre.

**Visualization:** Luigi Bianchi.

**Writing – original draft:** Giancarlo Ceccarelli, Gabriella d’Ettorre.

**Writing – review & editing:** Giancarlo Ceccarelli, Eugenio Nelson Cavallari, Antonio Ciardi.
